# Construction of Calcium Release Sites in Cardiac Myocytes

**DOI:** 10.3389/fphys.2012.00322

**Published:** 2012-08-20

**Authors:** Alexandra Zahradníková, Ivan Zahradník

**Affiliations:** ^1^Department of Muscle Cell Research, Institute of Molecular Physiology and Genetics, Slovak Academy of SciencesBratislava, Slovakia; ^2^Department of Biochemistry and Structural Biology, Institute of Molecular Biology, Slovak Academy of SciencesBratislava, Slovakia

**Keywords:** excitation-contraction coupling, cardiac myocyte, local calcium signaling, ryanodine receptors, calcium sparks

## Abstract

Local character of calcium release in cardiac myocytes, as defined by confocal recordings of calcium sparks, implies independent activation of individual calcium release sites based on ryanodine receptor (RyR) channel recruitment. We constructed virtual calcium release sites (vCRSs) composed of a variable number of RyR channels distributed in clusters in accordance with the experimentally observed cluster size distribution. The vCRSs consisted either of a single virtual calcium release unit (vCRU), in which all clusters shared a common dyadic space, or of multiple virtual calcium release units (CRUs) containing one cluster each and having separate dyadic spaces. We explored the stochastic behavior of vCRSs to understand the activation and recruitment of RyRs during calcium sparks. RyRs were represented by the published allosteric gating model that included regulation by cytosolic Ca^2+^ and Mg^2+^. The interaction of Mg^2+^ with the RyR Ca^2+^-binding sites and the refractory period of vCRSs were optimized to accord with the experimentally observed calcium dependence of calcium spark frequency. The Mg^2+^-binding parameters of RyRs that provided the best description of spark frequency depended on the number of RyRs assembled in the vCRSs. Adequate inhibitory effect of Mg^2+^ on the calcium dependence of RyR open probability was achieved if the vCRSs contained at least three clusters. For the distribution of the number of open RyRs in evoked calcium sparks to correspond to the experimentally observed distribution of spark calcium release fluxes, at least three clusters had to share a common virtual CRU, in which ∼3 RyRs open to form an average spark. These results reconcile the small cluster size and stochastic placement of RyRs in the release sites with the estimates of the amount of RyR protein, volume density of calcium release sites, and the size of calcium release sites in rat cardiac myocytes.

## Introduction

Calcium sparks (Cheng et al., 1993), the elementary calcium signals of excitation-contraction coupling in cardiac myocytes, result from activation of ryanodine receptors (RyRs). RyRs channels are clustered at the junctional membrane of the dyadic sarcoplasmic reticulum, thus forming the calcium releasing units (Sun et al., 1995). Stimulation of cardiac myocytes results in synchronous activation of calcium sparks (Cannell et al., 1994) by a calcium influx through the voltage-dependent calcium channels (DHPRs). Sparks occur also in the absence of stimulation under diastolic conditions (Cheng et al., 1993). Such resting calcium sparks are thought to constitute a significant component of calcium leak from the sarcoplasmic reticulum that regulates SR calcium content (Lukyanenko et al., 2001; Maier et al., 2003).

The relationships between RyR activity and calcium spark properties were partially cleared only recently. Specifically, the average number of RyRs that open during the spark was estimated to 2.7–20 (Bridge et al., 1999; Wang et al., 2004; Shkryl et al., 2012), and the calcium dependence of spark frequency was shown to be in line with the calcium dependence of RyR open probability (Lukyanenko et al., 2000; Gusev and Niggli, 2008; Zahradnikova et al., 2010b). The generally accepted image of calcium release units (CRUs) involved large clusters of tightly packed RyRs (Franzini-Armstrong et al., 1999; Soeller et al., 2007). Recent data indicate that both the RyR packing density and the size of CRUs is much smaller (∼14 RyRs per cluster; Baddeley et al., 2009) and highly variable, with RyRs arranged into clusters and superclusters (Baddeley et al., 2009; Hayashi et al., 2009). Quantitative composition of the superclusters has not been determined. It is not known as well, whether adjacent clusters share the same dyadic space and the same terminal cisterna. The observed amount of RyR protein (Bers and Stiffel, 1993) and the density of calcium release sites (Chen-Izu et al., 2006) indicate about 70 RyRs per release site (Soeller et al., 2007; Asghari et al., 2012).

The properties of calcium sparks should be governed by the single channel properties of RyRs, the size of RyR clusters, and the arrangement of clusters into release sites. Previously we have shown (Zahradnikova et al., 2010b) that the relationship between cytosolic calcium concentration and the frequency of spontaneous calcium sparks can be fully explained by gating of single, independent RyRs. Quantitative modeling of these processes with a uniform cluster size of 182 RyRs provided parameters of RyR gating in a very good accordance with both, the experimentally determined gating properties of the RyRs, and the calcium dependence of the frequency of spontaneous calcium sparks. It turned out, however, that changes in the size of the CRUs had a strong effect on the simulated calcium spark frequency (Zahradnikova et al., 2010b). Here we explored how the organization of RyRs into small and highly variable clusters can be brought into accordance with RyR gating.

Calcium release flux during calcium sparks has been shown previously to have quantal amplitudes due to opening of only a few RyRs on electrical stimulation (Wang et al., 2004). Quantal distribution of the spark release flux amplitudes is compatible with the model of CRU based on the RyR activation controlled by Mg^2+^/Ca^2+^ competition at the RyR activation sites (Zahradnikova et al., 2010b). According to this CRU model, designed with 182 RyRs per CRU, the Mg^2+^ ions occupy 92% of the activation sites on RyR channels and prevent explosive RyR activation by calcium. The low rate of Mg^2+^ dissociation primes only a few RyRs in the CRU for opening during the spark. Quantal distribution of release flux was shown to be compatible with smaller clusters as well (Zahradnikova et al., 2010a); however, quantitative correspondence with experiments has not been tested.

In this work, the model of calcium spark (Zahradnikova et al., 2010b) has been extended and used to explore the consequences of variability of RyR clusters and their arrangement into CRUs and calcium release sites that emerged from the recent ultrastructural studies (Baddeley et al., 2009; Hayashi et al., 2009).

## Materials and Methods

### Construction of RyR clusters, virtual CRUs, and virtual calcium release sites

The size of the simulated RyR clusters was determined, as previously described (Zahradnikova et al., 2010a), by drawing random numbers from the bi-exponential size distribution (Baddeley et al., 2009) with the following parameters:

(1) $$p\left(n_{\text{RyR}}\right)=0.113\;{\text{e}}^{-\frac{n_{\text{RyR}}}{3.2}}+0.032\;{\text{e}}^{-\frac{n_{\text{RyR}}}{20}},$$

where *n*_RyR_ is the number of RyR channels in the cluster (cluster size), and *p*(*n*_RyR_) is the probability that a randomly chosen cluster will have a size *n*_RyR_.

**Figure 1:**
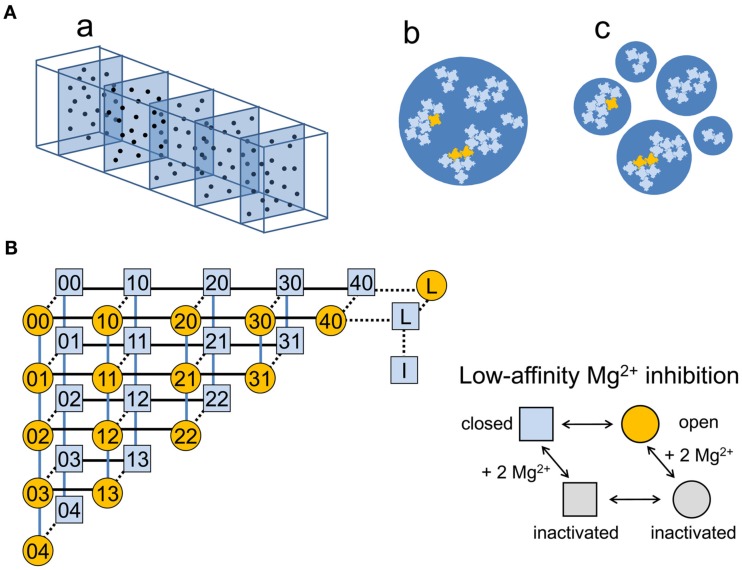
**Description of the model**. **(A)** Construction of the model. **(a)** The simulated volume of a cardiomyocyte (10 μm × 3.16 μm × 3.16 μm), consisting of 5 sarcomeres, each comprising 20 calcium release sites observable by the confocal microscope (blue dots). **(b)** An example of a virtual calcium release site consisting of one virtual calcium release unit (blue circle) comprising five RyR clusters. **(c)** An example of a virtual calcium release site consisting of five virtual calcium release units (blue circles), each comprising one RyR cluster. Closed RyRs are light blue, open RyRs are orange. **(B)** The gating scheme of the RyR channel. According to this allosteric homotetrameric RyR model (Zahradnikova et al., 2010b), the single channel properties of RyR channels are described by Eq. 3 and the parameter set given in Table 2. The light blue squares represent the closed states and the orange circles represent the open states of the channel. The first and the second numeral denote the number of calcium and magnesium ions, respectively, bound to the RyR Ca^2+^ activation sites. The black (horizontal) and the blue (vertical) lines indicate the calcium-dependent and the magnesium-dependent channel transitions, respectively. The transitions independent of ion binding/unbinding are depicted as dashed lines. All transitions between channel states are reversible and obey the principle of detailed balance. L stays for the low activity L-mode states, and I stays for the inactivated state. Inactivation of the channel by Mg^2+^ ions binding to the low-affinity inhibition site is depicted at the right.

A predefined number (1, 2, 3, 5, or 10) of individual RyR clusters was randomly combined into virtual calcium release units (vCRUs), which were considered to share a common dyadic space, i.e., the RyRs in a single vCRU sensed the same cytosolic and luminal environment (Figure 1Ab). One such vCRU was assumed to form a virtual calcium release site (vCRS). Alternatively, a predefined number of vCRUs (1, 2, 3, 5, or 10) consisting of a single cluster were combined into a vCRS. In this case, individual vCRUs constituting the vCRS were considered to behave independently, i.e., to have separate dyadic spaces (Figure 1Ac).

### Frequency of spontaneous calcium sparks

The frequency of spontaneous calcium sparks, *F*_spark_, was estimated according to Zahradnikova et al. (2010b):

(2) $$F_{\text{spark}}=\;\frac{n_{\text{vCRS}}}{t_{\text{rd}}+t_{\text{rp}}+\frac{\tau_{\text{O}}\left(1/P_{\text{O}}-1\right)}{n_{\text{RyR}}}},$$

where *P*_O_ is RyR open probability, *n*_vCRS_ is the number of vCRSs per 100 μm of a random longitudinal line scan, *t*_rd_ is the release duration, *t*_rp_ is the refractory period, τ_O_ is the RyR open time, and *n*_RyR_ is the average number of RyRs present in the calcium release sites. The values of the parameters are given in Table 1.

**Table 1:** **Parameters of the spark frequency model**.

Parameter	Value
τ_O_ (ms)	15
*t*_rd_ (ms)	15
*t*_rp_ (ms)	Fitted parameter
*n*_RyR_	Variable
*n*_vCRS_	36

*τ_O_ – mean RyR open time*.

**t*_rd_ – release duration*.

**t*_rp_ – refractory period for calcium release*.

**n*_RyR_ – mean/median number of RyR channels in the calcium release site*.

**n*_vCRS_ – the number of calcium release sites per 100 μm of a random longitudinal scan*.

The steady-state RyR open probability, *P*_O_, was expressed by the aHTG model of RyR gating (Zahradnikova et al., 2010b), in which the transitions between states differing in the occupation of the activation sites are described by the scheme in Figure 1B, and the channel is additionally regulated by an independent Mg^2+^ inhibition site. Thus, the open probability of the RyR is:

(3) $$\begin{array}{c} P_{\text{O}}=(K_{\text{CL}}K_{\text{CL}}({\left[\text{Ca}\right]}^{4}f_{\text{Mg}}^{4}K_{\text{Ca}}^{4}\left(f_{\text{Ca}}^{4}K_{\text{O}00}+K_{\text{OL}}\right)\\ \;+4\left[\text{Ca}\right]f_{\text{Ca}}^{3}f_{\text{Mg}}K_{\text{Ca}}^{3}K_{\text{Mg}}K_{\text{OL}}{\left(f_{\text{Mg}}K_{\text{Mg}}+\left[\text{Mg}\right]\right)}^{3}\\ \;+f_{\text{Ca}}^{4}K_{\text{Ca}}^{4}K_{\text{OL}}{\left(f_{\text{Mg}}K_{\text{Mg}}+\left[\text{Mg}\right]\right)}^{4}\\ \;+4{\left[\text{Ca}\right]}^{3}f_{\text{Ca}}f_{\text{Mg}}^{3}K_{\text{Ca}}K_{\text{Mg}}^{3}K_{\text{OL}}\left(f_{\text{Mg}}K_{\text{Mg}}+4\left[\text{Mg}\right]\right)\\ \;+6{\left[\text{Ca}\right]}^{2}f_{\text{Ca}}^{2}f_{\text{Mg}}^{2}K_{\text{Ca}}^{2}K_{\text{Mg}}^{2}K_{\text{OL}}(f_{\text{Mg}}^{2}K_{\text{Mg}}^{2}+4f_{\text{Mg}}K_{\text{Mg}}\left[\text{Mg}\right]\\ \;+{\left[\text{Mg}\right]}^{2})))/({\left[\text{Ca}\right]}^{4}f_{\text{Mg}}^{4}K_{\text{Mg}}^{4}(K_{\text{CL}}K_{\text{CI}}K_{\text{OL}}\\ \;+f_{\text{Ca}}^{4}K_{\text{O}00}(1+K_{\text{CI}}\left(1+K_{\text{CL}}+K_{\text{CL}}K_{\text{OL}}))\right)\\ \;+4\left[\text{Ca}\right]f_{\text{Ca}}^{3}f_{\text{Mg}}K_{\text{Ca}}^{3}K_{\text{CL}}K_{\text{CI}}K_{\text{Mg}}K_{\text{OL}}(f_{\text{Mg}}^{3}K_{\text{Mg}}^{3}\left(1+f_{\text{Ca}}K_{\text{O}00}\right)\\ \;+3f_{\text{Mg}}^{2}K_{\text{Mg}}^{2}\left(1+f_{\text{Ca}}f_{\text{Mg}}K_{\text{O}00}\right)\left[\text{Mg}\right]\\ \;+3f_{\text{Mg}}K_{\text{Mg}}\left(1+f_{\text{Ca}}f_{\text{Mg}}^{2}K_{\text{O}00}\right){\left[\text{Mg}\right]}^{2}\\ \;+\left(1+f_{\text{Ca}}f_{\text{Mg}}^{3}K_{\text{O}00}\right){\left[\text{Mg}\right]}^{3})\\ \;+f_{\text{Ca}}^{4}K_{\text{Ca}}^{4}K_{\text{CLKCI}}K_{\text{OL}}(f_{\text{Mg}}^{4}K_{\text{Mg}}^{4}\left(1+K_{\text{O}00}\right)\\ \;+4f_{\text{Mg}}^{3}K_{\text{Mg}}^{3}\left(1+f_{\text{Mg}}K_{\text{O}00}\right)\left[\text{Mg}\right]\\ \;+6f_{\text{Mg}}^{2}K_{\text{Mg}}^{2}\left(1+f_{\text{Mg}}^{2}K_{\text{O}00}\right){\left[\text{Mg}\right]}^{2}\\ \;+4f_{\text{Mg}}K_{\text{Mg}}\left(1+f_{\text{Mg}}^{3}K_{\text{O}00}\right){\left[\text{Mg}\right]}^{3}+\left(1+f_{\text{Mg}}^{4}K_{\text{O}00}\right){\left[\text{Mg}\right]}^{4})\\ \;+4{\left[\text{Ca}\right]}^{3}f_{\text{Ca}}f_{\text{Mg}}^{3}K_{\text{Ca}}K_{\text{CL}}K_{\text{CI}}K_{\text{Mg}}^{3}K_{\text{OL}}(2\left[\text{Mg}\right]\\ \;+f_{\text{Mg}}\left(K_{\text{Mg}}+f_{\text{Ca}}^{3}K_{\text{Mg}}K_{\text{O}00}+f_{\text{Ca}}^{3}K_{\text{O}00}\left[\text{Mg}\right]\right))\\ \;+6{\left[\text{Ca}\right]}^{2}f_{\text{Ca}}^{2}f_{\text{Mg}}^{2}K_{\text{Ca}}^{2}K_{\text{CL}}K_{\text{CI}}K_{\text{Mg}}^{2}K_{\text{OL}}4f_{\text{Mg}}K_{\text{Mg}}\left[\text{Mg}\right]+{\left[\text{Mg}\right]}^{2}\\ \;+f_{\text{Mg}}^{2}(K_{\text{Mg}}^{2}\left(1+f_{\text{Ca}}^{2}K_{\text{O}00}\right)+2f_{\text{Ca}}^{2}K_{\text{Mg}}K_{\text{O}00}\left[\text{Mg}\right]\\ \;+f_{\text{Ca}}^{2}K_{\text{O}00}{\left[\text{Mg}\right]}^{2}))).K_{\text{I}}^{2}/\left(K_{\text{I}}^{2}+{\left[\text{Mg}\right]}^{2}\right), \end{array}$$

where [Ca] and [Mg] are the free cytosolic concentrations of Ca^2+^ and Mg^2+^, and the equilibrium constants *K*_x_ and allosteric factors *f*_x_ are described and the values of the parameters are given in Table 2.

**Table 2:** **Parameters of RyR gating**.

Parameter	Value
*K*_Ca_ (μM)	0.6
*K*_Mg_ (μM)	Fitted parameter
*f*_Ca_	0.046
*f*_Mg_	Fitted parameter
*K*_O0_	10 800
*K*_OL_	0.5
*K*_CL_	0.89
*K*_CI_	3.0
*K*_I_ (μM)	760
[Mg] (mM)	1.0

**K*_Ca_ – calcium dissociation constant of the ion-free closed state C00*.

**K*_Mg_ – magnesium dissociation constant of the ion-free closed state C00*.

**K*_O0_ – dissociation constant of the ion-free open state O00*.

**f*_Ca_ – allosteric factor coupling calcium binding to channel opening*.

**f*_Mg_ – allosteric factor coupling magnesium binding to channel opening*.

**K*_OL_ – dissociation constant of the L-mode open state OL*.

**K*_CL_ – dissociation constant of the L-mode closed state CL*.

**K*_CI_ – dissociation constant of the inactivated state I*.

**K*_I_ – inhibitory constant of Mg^2+^ ions at the low-affinity inhibition site*.

*[Mg] – free Mg^2+^ concentration*.

Calcium spark frequency of a set of random RyR clusters was simulated using the formula

(4) $$F_{\text{spark}}=\;\frac{n_{\text{vCRS}}}{N}\overset{N}{\underset{i=1}{\sum }}\frac{1}{t_{\text{rd}}+t_{\text{rp}}+\frac{\tau_{\text{O}}\left(1/P_{\text{O}}-1\right)}{{\left(n_{\text{RyR}}\right)}_{i}}},$$

derived from Eq. 2 for non-uniform cluster sizes, where *N* is the number of simulated vCRSs (*n*_RyR_)*_i_* is the number of RyRs in the *i*^th^ simulated vCRS and the other symbols were explained previously.

### The number of open RyRs

The distribution of the number of open RyRs in a virtual CRU was calculated using the binomial equation (Zahradnikova et al., 2010b):

(5) $$P\left(n_{\text{O}}\right)=\left(\;\begin{array}{c} n_{\text{RyR}}-1\\ n_{\text{O}}-1 \end{array}\right)\;{\left(p_{\text{A}}\right)}^{n_{\text{O}}-1}-\;{\left(1-p_{\text{A}}\right)}^{n_{\text{RyR}}-n_{\text{O}}+1},$$

where *n*_RyR_ is the number of RyRs in the given virtual CRU, *n*_O_ is the number of activated/open RyRs in the vCRU, and *P*(*n*_O_) is the probability that *n*_O_ out of *n*_RyR_ RyRs will be open during the spark.

The values of *p*_A_ that optimized the distribution of the number of RyRs for the whole set of simulated CRUs were determined by minimizing the sum of squares of the differences between the predicted probabilities and those observed by Wang et al. (2004):

(6) $$\chi^{2}=\frac{\overset{n}{\underset{i=1}{\sum }}{\left(P\left(i\right)-P_{i}\right)}^{2}}{n-1},$$

where *i* is the number of open RyRs, *P*(*i*) was determined by applying Eq. 4 to all vCRUs, *P_i_* is the probability of occurrence of the *i*th quantal level, i.e., the number of open RyRs, determined by Wang et al. (2004), and *n* is the number of RyRs in the largest of the simulated vCRUs. The goodness of fit for each simulation was evaluated by the χ^2^ test (Press et al., 1992), using the method of Landau and Páez (1997) to determine experimental variance, as described previously (Zahradnikova et al., 1999).

### Parameters of Mg^2+^ unbinding

The parameters of Mg^2+^ unbinding from the activation site of the RyR channel were determined from activation probability, *p*_A_, as described in Zahradnikova et al. (2010b). Briefly, for each simulation, the dependence of the probability of Mg^2+^ dissociation (*p*_dis_) during the spark on *p*_A_ was calculated from the steady-state open probabilities of all ionic forms of RyR, defined by the number of bound Mg^2+^ and Ca^2+^ ions, using the parameters of the aHTG model determined for the respective simulation. The value of *p*_dis_ together with an estimate of the time to the peak release flux (TTP) then yielded an estimate of Mg^2+^ dissociation rate, $k_{\text{dis}}^{\text{Mg}}$, as described previously (Zahradnikova et al., 2010b):

(7) $$k_{\text{dis}}^{\text{Mg}}=-\frac{\ln\left(1-p_{\text{dis}}\right)}{\text{TTP}},$$

where all parameters have already been explained.

Derivation of equations was performed in Mathematica (Ver. 8.0, Wolfram Research). Analyses were performed in Mathematica (Ver. 8.0, Wolfram Research) and Origin (Ver. 8.0 SR6, OriginLab).

## Results

### Construction of the model

A model of a segment of a cardiac myocyte, containing 100 calcium release sites (Figure 1Aa) was designed as follows: A set of 1000 RyR clusters containing altogether 14095 RyRs was generated by randomly drawing 1000 numbers from a bi-exponential distribution of cluster sizes (Eq. 1). The resulting set of random “RyR clusters” was randomly recombined into two sets of vCRSs with the same size distribution. In the first set, each vCRS contained a single supercluster CRU, into which the RyR clusters were combined, as depicted in Figure 1Ab. In the second set, the vCRS contained RyR clusters of the same size, each cluster forming a separate single-cluster CRU (Figure 1Ac).

**Figure 2:**
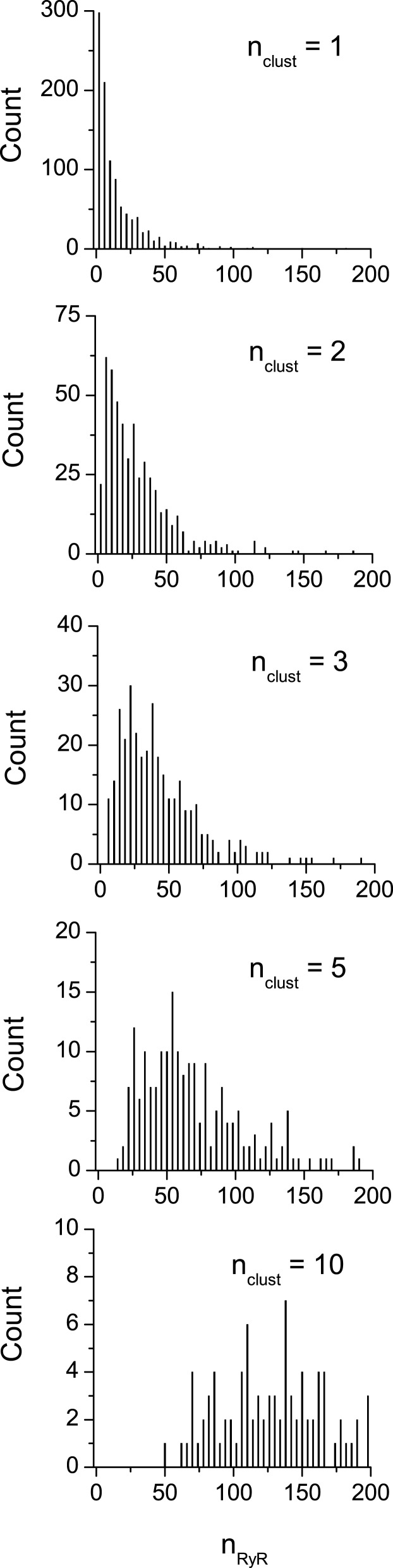
**Simulation of RyR distribution into clusters and virtual calcium release units (vCRUs)**. Distribution of the size of release units (in number of RyRs per release unit, *n*_RyR_) composed of (top to bottom) 1, 2, 3, 5, and 10 random clusters. The number of RyRs in clusters was distributed bi-exponentially according to Baddeley et al. (2009).

The size distribution of the vCRSs containing 1, 2, 3, 5, or 10 clusters is shown in Figure 2 and quantitatively characterized in Table 3. For vCRSs composed of a single cluster, the size distribution was in a good agreement with the experimental data (Baddeley et al., 2009). In multi-cluster vCRSs, the minimum number of RyRs ranged from 1 to 51 and their maximum number ranged from 180 to 271, respectively, while the mean size ranged from 14 to 141 RyRs and the median size ranged from 7 to 136.5 RyRs (Table 3). With increasing number of clusters per vCRS, the size distribution of the vCRSs gradually changed from bi-exponential to nearly Gaussian. With increasing number of clusters per vCRS, the total number of vCRSs decreased (from 1000 to 100), which was reflected in the increased variance in the size distribution histograms. When the total number of the generated vCRSs was more than 100, i.e., for *n*_clust_ < 10, a set of 100 vCRSs was randomly selected for further simulation.

**Table 3:** **Composition of virtual calcium release units**.

*n*_clust_	${\bar{n}}_{\text{RyR}}$	${ñ}_{\text{RyR}}$	Range
1	14.1 ± 18.0	7	1–180
2	28.2 ± 25.6	21	2–185
3	42.3 ± 29.8	36	4–189
5	70.5 ± 39.8	60	13–214
10	141.0 ± 51.2	136.5	51–271

**n*_clust_ – number of clusters per vCRU*.

*${\bar{n}}_{\text{RyR}}$ – mean ± SD of the number of RyRs per vCRU*.

*${ñ}_{\text{RyR}}$ – median number of RyRs per vCRU*.

*Range – minimum and maximum number of RyRs per vCRU*.

In all models it was assumed that all RyRs in a CRU are equivalent in the sense that they share the cytosolic as well as the luminal environment, and therefore they have the same steady-state open probability, *p*_O_, and the same probability of being activated during a spark, *p*_A_. In other words, the cytosolic and luminal environments of the RyR channels in a single CRU were considered as ideally mixed compartments. The vCRSs with multiple CRUs (Figure 1Ac) emulate the effect of insufficient spatial resolution of confocal microscopes. The probability of RyR activation in any CRU was not affected by the activation of RyRs in the remaining CRUs. Individual vCRSs were fully independent as well.

### Calcium dependence of spark frequency

The model of calcium spark frequency is based on the idea of triggering of a spark by the first spontaneous opening of single RyR channel in the calcium release site, arising from the resting open probability of RyRs (Zahradnikova et al., 2010b). In this model, all RyR channels in the vCRS are under resting conditions before activation of a calcium spark. The activation of a calcium spark corresponds to the stochastic opening of the first RyR in the vCRS. As a result, the frequency of sparks depends only on the total number of RyRs in the vCRS (Eq. 2) and not on the number and composition of vCRUs within the vCRS.

**Figure 3:**
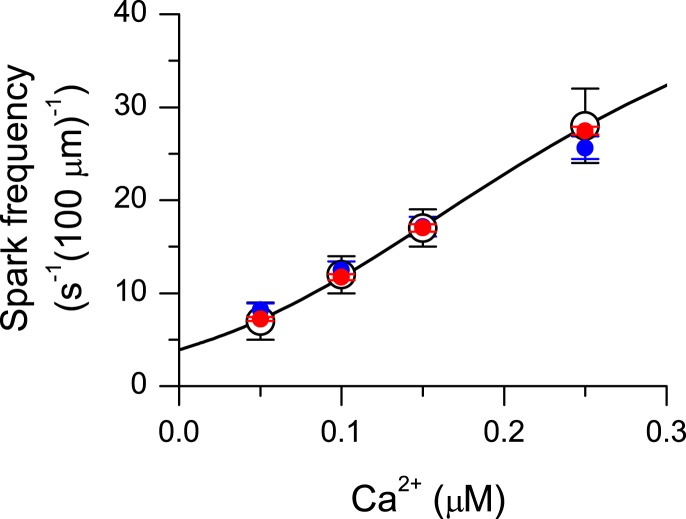
**Approximation of the calcium dependence of spark frequency**. Open symbols with error bars represent data on permeabilized myocytes (Lukyanenko and Gyorke, 1999). The two indistinguishable lines represent the best fits by Eq. 2 with parameters shown in Tables 1, 2, and 4 for the optimal aHTG model for vCRSs composed of 1 and 10 random clusters with the size distribution shown in Figure 2. Small red and blue symbols represent spark frequency simulated using Eq. 4 for a set of 100 random vCRUs composed of 1 and 10 clusters.

To adjust the constructed segments to real myocytes, we tuned their calcium dependence of calcium spark frequency to that measured by Lukyanenko and Gyorke (1999). The experimental calcium dependence of spark frequency (open symbols, Figure 3) was approximated by Eq. 2, into which Eq. 3 was substituted for *P*_O_. To obtain the best approximation of the data by the models, the parameters describing interaction of Mg^2+^ with the RyR activation sites (i.e., *K*_Mg_ and *f*_Mg_) and the refractory period (*t*_rp_) were optimized. A good agreement between the theoretical calcium dependence (Eq. 2) and experimental data could be obtained with the mean $\left({\bar{n}}_{\text{RyR}}\right)$ as well as with the median $\left({ñ}_{\text{RyR}}\right)$ number of RyRs per vCRS (albeit with slightly different optimized values of *K*_Mg_, *f*_Mg_, and *t*_rp_). The remaining gating parameters were kept at their original values, estimated by an independent method (Zahradnik et al., 2005). The adequacy of the optimized parameters *K*_Mg_, *f*_Mg_ and *t*_rp_ was then tested by comparing spark frequency in the set of vCRSs, calculated using Eq. 4, with the experimental data. It turned out that to obtain a good agreement between the simulation with each set of vCRSs and the experimental data, the median (small symbols in Figure 3) but not the average of the number of RyRs per vCRS had to be used as the descriptor of vCRS size in Eq. 2. The use of the mean number of RyRs per vCRS led to disagreement between the experimentally measured and the simulated calcium spark frequencies (not shown), most probably due to the skewness of the distribution of vCRS sizes. This was especially apparent at small vCRS sizes, in which there was a large difference between ${\bar{n}}_{\text{RyR}}$ and ${ñ}_{\text{RyR}}$ (Table 3). The median number of RyRs was an excellent predictor even for the case of the highly asymmetric bi-exponential distribution at *n*_clust_ = 1.

**Figure 4:**
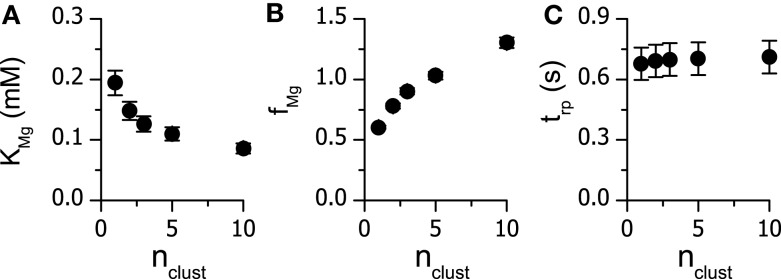
**The dependence of the fitted parameters on the number of clusters in the virtual calcium release sites**. Optimized values of *K*_Mg_ **(A)**, *f*_Mg_ **(B)** and *t*_rp_ **(C)** are given with the standard errors of estimate.

The characteristics of Mg^2+^ binding to the RyR for vCRS models containing a different number of clusters (*n*_clust_) were very sensitive to *n*_clust_ (Figures 4A,B; Table 4). The value of *K*_Mg_ was smaller by more than 50% when *n*_clust_ was increased from 1 to 10. For *n*_clust_ > 3 the optimal allosteric factor was larger than 1, as estimated previously for CRSs containing 182 RyRs (Zahradnikova et al., 2010b), implying a small negative allosteric effect of Mg^2+^ on RyR open probability. For *n*_clust_ ≤ 3, the value of *f*_Mg_ was less than 1, implying a small positive allosteric effect of Mg^2+^ on RyR open probability. The estimate of the refractory period *t*_rp_ was not sensitive to changes in *n*_clust_ (Figure 4C).

**Table 4:** **Mg^2^^+^ binding parameters of the RyR channel**.

*n*_vCRU_	*n*_clust_	*K*_Mg_ (μM)	*f*_Mg_
1	1	190 ± 20	0.60 ± 0.01
1	2	150 ± 20	0.78 ± 0.02
1	3	130 ± 10	0.90 ± 0.03
1	5	110 ± 10	1.03 ± 0.03
1	10	86 ± 8	1.30 ± 0.04
1	1	190 ± 20	0.60 ± 0.01
2	1	150 ± 20	0.78 ± 0.02
3	1	130 ± 10	0.90 ± 0.03
5	1	110 ± 10	1.03 ± 0.03
10	1	86 ± 8	1.30 ± 0.04

**n*_vCRU_ – number of vCRUs per virtual calcium release site*.

**n*_clust_ – number of clusters per vCRU*.

**K*_Mg_ – Mg^2+^ dissociation constant of the ion-free closed state C00*.

**f*_Mg_ – allosteric factor coupling Mg^2+^ binding to channel opening*.

**Figure 5:**
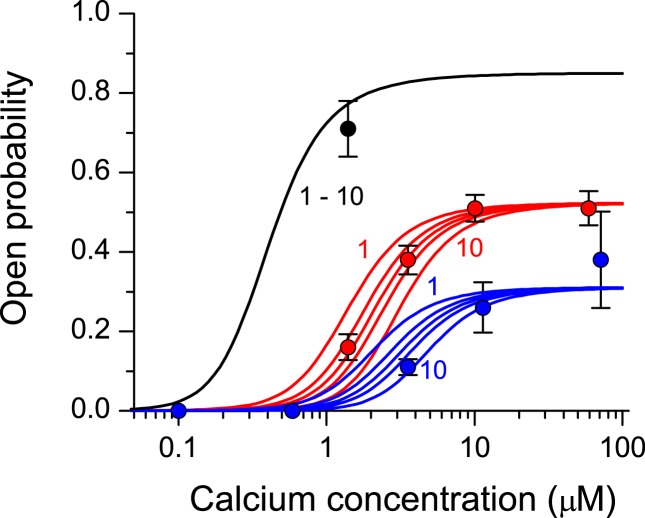
**Approximation of the calcium dependence of RyR open probability at different cytosolic Mg^2+^ concentrations**. Lines depict theoretical open probability of RyRs with parameter values (Tables 2 and 4) optimized to fit the calcium dependence of spark frequency for vCRSs composed of 1, 2, 3, 5, and 10 clusters in the absence (black) and in the presence of 0.6 (red, left to right for increasing *n*_clust_) or 0.9 mM Mg^2+^ (blue, left to right for increasing *n*_clust_).

The Mg^2+^-binding properties of the RyR had a significant impact on RyR open probability in the whole range of cytosolic Ca^2+^, as documented in Figure 5. Here the simulated calcium dependence of open probability is shown superimposed on the published experimental data obtained in the presence of 3 mM ATP and defined concentrations of free Mg^2+^ (Zahradnikova et al., 2003). The lower *f*_Mg_ and the higher *K*_Mg_ values at *n*_clust_ of 1 and 2 led to a significantly weaker inhibition of RyR open probability than experimentally observed, especially at higher [Mg^2+^]. The best correspondence between data and simulation occurred for *n*_clust_ = 3–10.

### Distribution of calcium release flux

The distribution of spark calcium release flux was calculated for all simulated vCRSs using Eq. 5. When vCRSs were composed of a single multi-cluster vCRU, it was assumed that after opening of the first RyR in the vCRS, all remaining RyRs in the vCRS have the same probability *p*_A_ of becoming open. When the vCRSs were composed of several single-cluster vCRUs, it was assumed that after opening of the first RyR in one vCRU, all remaining RyRs in that vCRU have the same probability *p*_A_ of becoming open, while the RyRs in the remaining vCRUs have the resting probability of becoming open, i.e., they are not affected by the RyR opening in the other vCRUs of the same vCRS. Since under all tested conditions the resting open probability of the RyR was ∼1 × 10^−4^, the probability that a resting RyR from another cluster of the same vCRS will open during the spark was less than 0.0035, which would result in a 0.35% excess of RyR openings, less than the estimated error of approximation of the data (Wang et al., 2004) by the best model (0.95%). Therefore the contribution of resting RyR openings to the distributions of calcium release flux was not taken into account.

**Figure 6:**
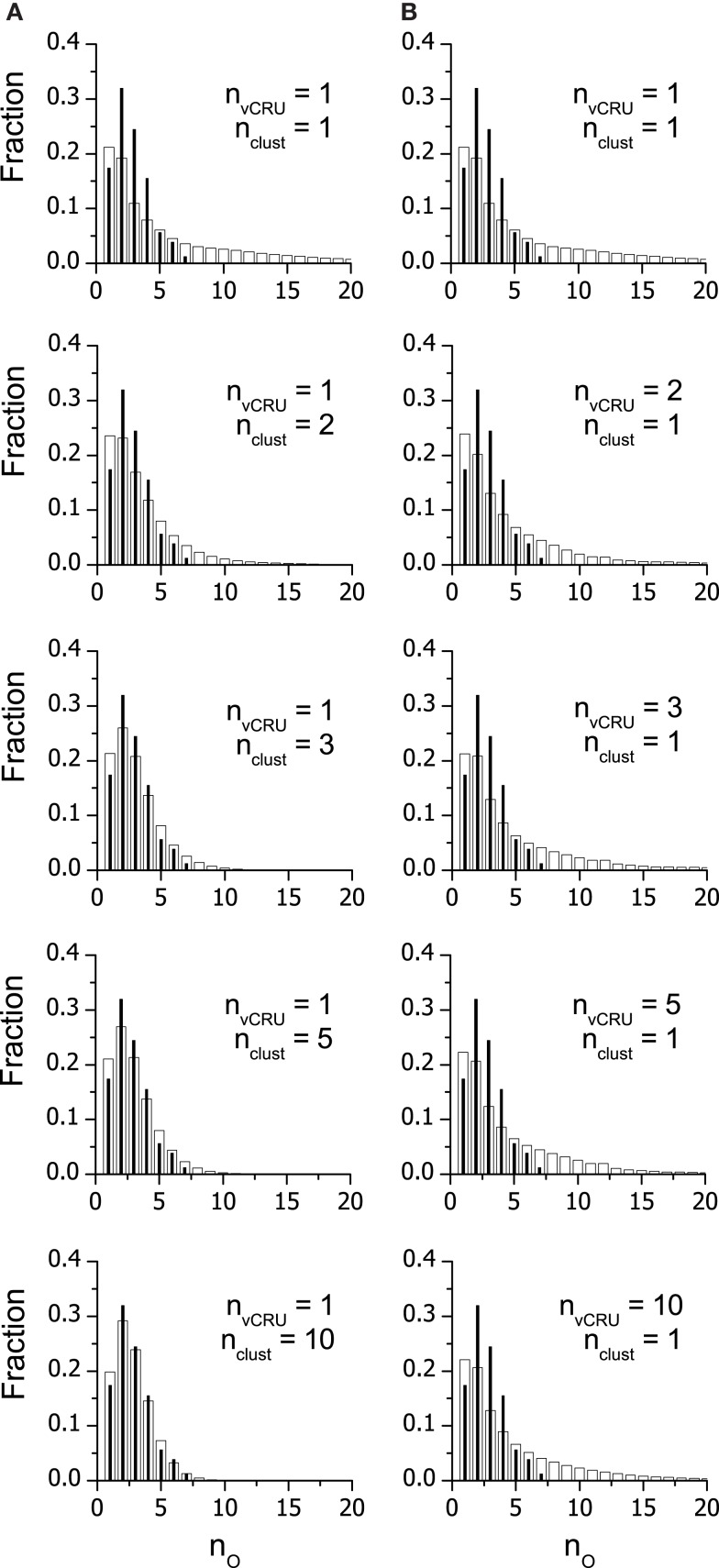
**Approximation of the distribution of calcium release fluxes with the theoretical distribution of the number of open RyRs in sparks**. Black bars represent the probability distribution of spark fluxes recorded under control conditions (Wang et al., 2004, their Figure 2) expressed in units of the elementary quanta of calcium release flux; white bars represent the probability distribution of the number, *n*_O_, of RyRs open during spark, calculated using Eq. 2 for sets of simulated vCRUs formed of (top to bottom) 1, 2, 3, 5, and 10 random clusters with size distribution from Figure 2, respectively. **(A)** vCRSs composed of one single- or multicluster vCRU, **(B)** vCRSs composed of one or more single-cluster vCRUs.

The activation probability *p_A_* for each set of vCRSs with a given *n*_clust_ was estimated by approximating the distribution of *n*_O_ (Eq. 5) to the experimental data published by Wang et al. (2004). The optimal distributions are shown in Figure 6 together with the experimental distribution. The values of *p*_A_ providing the optimal fit and the resulting average numbers of open RyRs per spark are given in Table 5. For vCRSs composed of a single multi-cluster vCRU (Figure 6A), it is apparent that for low *n*_clust_ of 1 or 2 the theoretical distributions did not describe the data adequately (χ^2^ test, *p* < 0.001). A satisfactory description of the data was obtained for *n*_clust_ = 3–10 (χ^2^ test, *p* > 0.1). If the vCRS was composed of a set of single-cluster, non-interacting vCRUs, the theoretical distributions of the spark calcium release flux did not depend on *n*_clust_ (Figure 6B) and were not adequate (χ^2^ test, *p* < 0.001).

**Table 5:** **Parameters of *n*_O_ distributions**.

*n*_vCRU_	*n*_clust_	${\bar{n}}_{\text{O}}$	*p*_A_
1	1	4.10	0.297
1	2	3.79	0.085
1	3	3.03	0.040
1	5	2.96	0.028
1	10	2.79	0.013
1	1	4.10	0.297
2	1	3.79	0.219
3	1	3.90	0.292
5	1	4.00	0.286
10	1	3.92	0.279

**n*_vCRU_ – number of vCRUs per virtual calcium release site*.

**n*_clust_ – number of clusters per vCRU*.

*${\bar{n}}_{\text{O}}$ – mean number of RyR openings per spark*.

**p*_A_ – probability of recruitment of a RyR channel into open state*.

### The rate of Mg^2+^ dissociation from the activation sites

**Figure 7:**
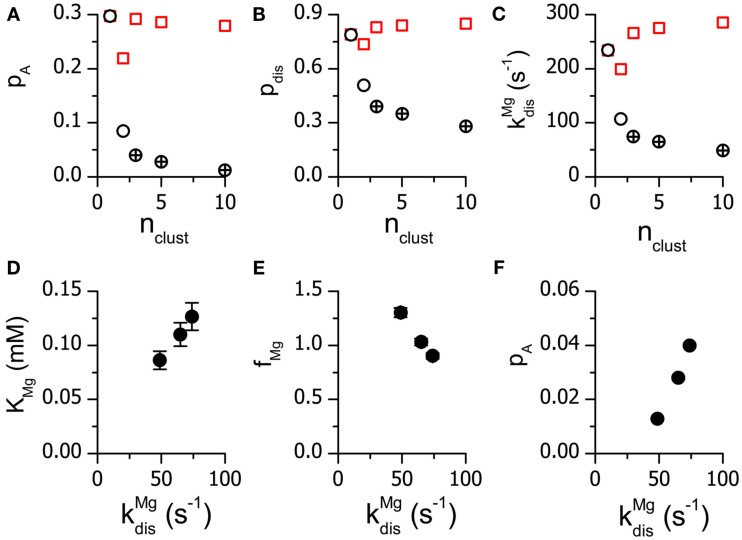
**The dependence of RyR kinetic parameters on the cluster composition of vCRSs**. **(A–C)** The dependence of *p*_A_, *p*_dis_, and $K_{\text{dis}}^{\text{Mg}}$on the number of clusters in the vCRS. Circles: vCRSs composed of a single multi-cluster vCRU. Squares: vCRSs composed of several single-cluster vCRUs. Crossed symbols: values from models with *n*_clust_ ≥ 3 and *n*_vCRU_ = 1 that match the experimental data (Figure 6). **(D–F)** The dependence of *K*_Mg_, *f*_Mg_, and *p*_A_ on $K_{\text{dis}}^{\text{Mg}}$ for vCRUs with different number of clusters.

If the clusters in the vCRS formed a single vCRU, the probability of recruitment of additional RyRs after activation of the first RyR (*p*_A_) decreased with increasing *n*_clust_. We tested the extent to which these changes were due to the changes in the dissociation of Mg^2+^ from the activation sites. The estimated values of *p*_A_ (Figure 7A) were converted into values of probability of Mg^2+^ dissociation from the activation site during the spark, *p*_dis_ (Figure 7B), using the procedure described in the Supplemental data of Zahradnikova et al. (2010b). These were further converted to Mg^2+^ dissociation rates (Figure 7C), assuming that Mg^2+^ dissociation has to occur not later than at the time to the peak of the spark (6.67 ms, Eq. 6). The values of all three parameters decreased with *n*_clust_ (circles); the models that did not correspond to the observed distributions of calcium release flux (Figure 6A) provided considerably higher values (open symbols) than the accepted models (crossed symbols). If the clusters in the vCRS formed separate vCRUs, the probability of recruitment of additional RyRs into sparks after activation of the first RyR did not change considerably with *n*_clust_ (squares in Figure 7). However, neither of these models corresponded to the experimental distributions of calcium release flux (Figure 6B).

Additionally, variation of the number of clusters present in one vCRS requested changes in the calcium dependence of RyR activation to follow the calcium dependence of RyR open probability at different Mg^2+^ concentrations (Figure 5). Specifically, with increasing number of clusters per vCRS, the RyRs had to undergo stronger inhibition by Mg^2+^ at their activation sites (smaller *K*_Mg_ and larger *f*_Mg_). The extent, to which the changes in *K*_Mg_, *f_Mg_*, and *p*_A_ are due to changes of $k_{\text{dis}}^{\text{Mg}},$ is examined in Figures 7D–F for vCRSs that were accepted by the χ^2^ test. The increase in $k_{\text{dis}}^{\text{Mg}}$translates into a proportional increase of *K*_Mg_ and a decrease of *f*_Mg_. The value of *p_A_* is very steeply dependent on $k_{\text{dis}}^{\text{Mg}}.$

## Discussion

This study reveals that not only the gating of RyR channels, but also their organization into release sites plays a role in the cardiac mechanism of excitation-contraction coupling. This would be difficult to reveal experimentally.

The relationships between the organization of RyRs into calcium release sites and the properties of calcium sparks were explored using extensions of our previously described models of calcium spark and RyR gating (see Materials and Methods). The results have shown that for a range of calcium release site sizes $\left({\bar{n}}_{\text{RyR}}=42.3-141;\;{ñ}_{\text{RyR}}=36-136.5\right)$ and size distributions, a model of calcium spark formation based on independent RyRs provides a quantitative description of the calcium dependence of calcium spark frequency (Figure 3). The two alternative hypotheses, that the confocally resolved volume comprises either a single vCRU containing three or more clusters, or several small vCRUs containing one cluster each, provided identical results, since all RyRs present in the confocally resolved volume are indistinguishable under resting conditions.

Moreover, the quantal distribution of spark calcium release flux in these CRUs is in a good agreement with the experimental data (Figure 6), if the RyR clusters in the calcium release sites are organized into a single CRU, that is, all RyRs of the CRS experience the same conditions modulating their steady-state open probability and probability of activation.

### The relation between CRS distribution, calcium spark frequency and RyR open probability

The parameters of Mg^2+^ interaction with RyR channels (*K*_Mg_ and *f*_Mg_) were optimized for different sets of vCRS sizes. A satisfactory fit of calcium spark frequency could be obtained for all vCRSs at the expense of substantial changes in the parameters *K*_Mg_ and *f_Mg_* (Table 4).

The Ca^2+^ dependence of open probability of RyRs that provided the correct calcium dependence of calcium spark frequency was compared with experimental data at different Mg^2+^ concentrations (Figure 5). The simulated inhibitory effect of Mg^2+^ on the calcium dependence of RyR open probability was in better accordance with the experimental observations for larger vCRS sizes (*n*_clust_ × *n*_vCRU_ ≥ 3). For smaller vCRSs, the correct calcium dependence of calcium spark frequency could be obtained only if RyRs inhibition by Mg^2+^ at the RyR activation sites was weaker (*K*_Mg_ = 150–190 μM) than experimentally estimated (61–100 μM; Gusev and Niggli, 2008; Zahradnikova et al., 2010b; Tencerova et al., 2012) except for Zahradnikova et al. (2003; 250 ± 150 μM).

### Quantal distribution of calcium release flux

Unequivocal results regarding the composition of vCRSs were provided by analysis of the quantal distribution of calcium release flux in evoked calcium sparks. The theoretical predictions of the models corresponded with experimental data (Wang et al., 2004) only for vCRSs consisting of a single larger vCRU (*n*_clust_ > 3). For vCRSs consisting of a single smaller vCRU (*n*_clust_ = 1, 2), and for vCRSs composed of up to ten small vCRUs (*n*_clust_ = 1), there was an excess of predicted sparks with high calcium release flux (*n*_O_ > 7) and a deficit of the predicted sparks with average calcium release flux (*n*_O_ = 2–3), which resulted in unacceptably high deviation of the model from the data. Since independent recruitment of RyRs after commencement of the spark, indicated by the binomial distribution of their calcium release flux, requires their equal access to the calcium trigger, all clusters in the vCRS had to communicate via the shared cytosolic/luminal environment, i.e., to consist of a single multi-cluster vCRU.

### The rates of Mg^2+^ dissociation from the RyR

The rate of Mg^2+^ dissociation obtained by optimizing the quantal distribution of calcium release flux was strongly dependent on the assumed vCRS size, and ranged from 48 to 224 s^−1^.A satisfactory accordance with experimental data was obtained only for vCRS size of 3–10 clusters, yielding $k_{\text{dis}}^{\text{Mg}}=48-74\;{\text{s}}^{-1}.$

These values are comparable with or higher than the rates of dissociation of Mg^2+^ from the Mg^2+^-parvalbumin complex (4–33 s^-1^; Permyakov et al., 1987) or from its complex with troponin-C or with the C-terminal fragment of troponin-C (5 and 0.7 s^−1^; Rosenfeld and Taylor, 1985). At the same time, they are somewhat lower than the rates of Mg^2+^ dissociation from ATP (150 s^−1^; Baylor and Hollingworth, 1998). A relatively low value of $k_{\text{dis}}^{\text{Mg}}$ may also be inferred from the strong effect of low Mg^2+^ concentrations on activation of RyR channels by brief Ca^2+^ stimuli (Zahradnikova et al., 2003). Therefore, the values of $k_{\text{dis}}^{\text{Mg}}$estimated for *n*_clust_ of 3–10 may be considered plausible.

## Conclusion

The modeling of the calcium release sites showed that RyRs with gating characteristics conforming to their behavior in lipid bilayers, and organized in clusters with size distribution revealed by experiments, provide a very satisfactory description of calcium spark properties in both, spontaneous and triggered sparks, collected in independent experiments. According to the best models, RyR clusters associate into CRUs composed of at least 3 clusters and containing at least 40 RyRs on average, which share the same dyadic space and environmental conditions. Moreover, these results reconcile the observed small cluster size and somewhat irregular placement of RyRs in the release sites with the previous estimates of the amount of RyR protein (Bers and Stiffel, 1993), volume density of calcium release sites (Chen-Izu et al., 2006), and the extent of calcium release in rat cardiac myocytes (Zahradnikova et al., 2010b).

## Conflict of Interest Statement

The authors declare that the research was conducted in the absence of any commercial or financial relationships that could be construed as a potential conflict of interest.
